# Communication Breakdown: Improving Communication Between Transplant Centers and Dialysis Facilities to Improve Access to Kidney Transplantation

**DOI:** 10.1016/j.xkme.2021.08.003

**Published:** 2021-08-24

**Authors:** Avrum Gillespie

**Affiliations:** Section of Nephrology, Hypertension, and Kidney Transplantation, Department of Internal Medicine, Lewis Katz School of Medicine, Temple University, Philadelphia, PA

Related article, p. 799

In the United States, although there is near-universal access to hemodialysis, there are many barriers to kidney transplantation.^1^ As a result, in-center hemodialysis is the most prevalent treatment for end-stage kidney disease.^2^ One of the goals of the Advancing American Kidney Health Initiative is to increase the number of Americans who receive a kidney transplant.^3^ With more than 500,000 Americans receiving maintenance in-center hemodialysis,^2^ the hemodialysis clinic, at which patients are receiving treatments for on average 11 hours per week and are surrounded by members of a multidisciplinary care team, is a logical place for interventions that increase access to kidney transplantation.

In this issue of *Kidney Medicine*, Browne et al^4^ ask both kidney transplant centers (N = 9) and dialysis facilities (N = 421) in the Southeastern United States participating in the Reducing Disparities in Access to Kidney Transplantation (RaDIANT) Regional Study and Network 6's Transplant Quality Improvement Project to identify observed barriers to kidney transplantation and potential interventions to overcome these barriers.

Browne et al identified a communication breakdown between transplant centers and dialysis facilities. Both transplant center staff and dialysis facility staff reported that if they communicated with each other more frequently, it would be easier for patients to access the kidney transplant waitlist; however, neither transplant center staff nor the dialysis facility staff suggested how communication could be improved. Notably, I agree with the authors that most of the barriers to the waitlist, such as help navigating the steps in the evaluation process, health literacy deficits, transportation barriers, and insufficient education, can be overcome by dialysis facilities partnering with transplant centers to improve communication and knowledge dissemination (Table 1).

**Table 1:** High-Yield Communication Interventions

Domain(s)	Transplant Center’s Role	Dialysis Facility’s Role
Improved modes of communication and coordination	Provide the dialysis facility with access to the electronic medical records so that the dialysis facility can track the progress of the transplant process, including current listing status; provide a hotline for the dialysis facilities to contact the transplant center with patient issues	Designate a team leader whose role it is to track patients’ progress through the transplant steps and patients’ current listing status; team leader notifies the transplant center about any changes in the patient’s condition
Logistics of completing the transplant evaluation process	Notify dialysis facility of upcoming transplant evaluation appointments; use satellite clinics and telemedicine when possible	All dialysis staff remind patients of upcoming appointments; help with transportation logistics when possible
Patient advocacy by the dialysis facility	Provide the dialysis facility transplant team leader access to the transplant center’s multidisciplinary listing committee meeting	Team leader advocates for the patient
Patient education and transplant interventions to improve access	Educating the dialysis facility staff about the transplant process so the dialysis staff can communicate effectively about the transplant process in a “train the trainer” model; collaborate with dialysis facility in providing multimedia educational material and transplant interventions	Every staff member should have a role in promoting and helping patients through the steps toward transplantation; collaborate with transplant center in distributing multimedia educational material and transplant interventions

*Note:* This table shows the high-yield communication interventions identified in the article by Browne et al^4^ and the domain(s) each intervention can be classified in, the transplant center’s role, and the dialysis facility’s corresponding role.

First, what must be addressed is how transplant centers and dialysis facilities communicate. In the digital age, some transplant centers have established online portals and apps for patients to track their progress through the transplant process; however, as identified in the survey, only half of the transplant centers directly communicated by telephone while a quarter of transplant centers relied on fax alone. This inability to properly transmit and convey information is further exacerbated because many of the hemodialysis facilities’ electronic medical records do not interface with the transplant centers’ electronic medical records.^5^ Additionally, in my experience, I found that speaking with another person on the telephone is far and away the most reliable way to confer complex information, as well as to avoid and clarify misunderstandings, and I recommend that all centers have such a hotline readily available for the dialysis facility staff and dialysis clinicians to call with questions.

Another simple intervention to increase access to transplantation proposed by the authors, which I have used in my own clinics, is the following: during the transplant evaluation process, both the potential recipients and the dialysis facility are notified of all upcoming transplant evaluation appointments. These appointments with various medical providers, which are part of the transplant candidacy evaluation, are often the points at which patients get “stuck” in the evaluation process.^6^ If dialysis facilities are also aware of the times of the appointments, they can frequently remind the patient, 3 times per week perhaps, of the upcoming appointments and explain the importance of the appointments and why the appointments are necessary.

This also may help address the barrier of health literacy that was identified by transplant centers. By incorporating the dialysis facility in the scheduling of appointments, the dialysis facility also can ensure that these appointments do not interfere with the patient’s dialysis schedule and the dialysis facility may even be able to assist with local transportation, which was another identified barrier.

Transportation logistics to and from dialysis is something that facilities deal with daily. Helping with transplant evaluation appointments will likely decrease the number of patients deemed ineligible because of missed appointments, which was a policy at most of the surveyed transplant centers.

Most surveyed dialysis facilities (79.1%) had a staff member within the multidisciplinary dialysis team whose role it is track patients’ progress through the transplant steps and patients’ current listing status. This is an essential role because patients may be confused about whether they are on the transplant list.^7^ Team leaders were usually the facility social worker whose training intersects medical and social problems. Nevertheless, having only 1 person in the clinic responsible for educating about and promoting transplantation is an unfair burden. Everyone within the facility should have a role in promoting and helping patients through the steps towards transplantation.^8^ This includes providing correct information about the transplant process and transplant outcomes and reminding patients about upcoming appointments. Every member of the team should be trained to communicate effectively about kidney transplantation. Additionally, dialysis staff should also correct incorrect or incomplete information about kidney transplantation that may come from other patients and members of the dialysis community.

One such program developed by Waterman et al^9^ teaches all members of the staff about the benefits and risk of transplantation and how to discuss it with patients. This can be challenging for hemodialysis staff, who often need to balance promoting transplantation among those who are potentially eligible for transplantation without disheartening patients who are ineligible, a particularly important point because dialysis facilities reported that high comorbidity burden was a common barrier to transplantation. Although increased education was identified by both transplant programs and hemodialysis facilities as important interventions to increase access to transplantation, education is not always enough to change attitudes and behaviors.

After experiencing and surviving the major life-changing event of initiating dialysis, patients may be reluctant to seek another major surgery and life-changing event. Although on average, transplantation leads to better outcomes, these outcomes are not guaranteed.^10^ Furthermore, within the dialysis facility, they may not have the opportunity to discuss transplantation with someone who had a successful transplant and may be disproportionately exposed to fellow patients who either do not want a kidney transplant, are ineligible for kidney transplantation, or had a failed kidney transplant.^8^ Thus, as Browne et al suggested, dialysis facilities need a “cheerleader” or, as I suggest, a squad of cheerleaders who motivate the patient to continue to pursue and complete the sometime arduous steps towards transplantation.^8^ Finally, with increased use of telemedicine and remote meetings, perhaps a member of the dialysis facility staff can participate in the kidney transplant listing committee meeting and advocate for candidate patients.

Although this study clearly identifies transplantation barriers for dialysis patients and readily actionable interventions, implementation remains the challenge. Educating and training dialysis facility staff requires a significant investment of time and money, including high-quality informational materials such as educational brochures and videos, and it is unclear whether transplant centers, dialysis providers, or dialysis regional network organizations should fund these development and dissemination efforts. However, with the Advancing American Kidney Health initiative mandating an increase in the number of kidney transplants and eligible patients on the waitlist^3^ and the implementation of new care models that include financial incentives for nephrologists and dialysis facilities to increase transplantation,^11^ funding necessary interventions is highly incentivized within the kidney community.

As a kidney community, we are mandated to increase the rate of kidney transplantation. To accomplish this, we must re-evaluate whether the role of dialysis facilities is solely to provide the best dialysis treatment possible or to provide the best outcomes for people living with kidney failure by also promoting and facilitating kidney transplantation. Is the next logical step for the dialysis units to help patients request a living donation through health care educator interventions^9^^,^^12^, ^13^, ^14^? This study by Browne et al clearly shows the barriers that need to be addressed in improving access to kidney transplantation and now it is up to the nephrology and transplant community to act. I look forward to future interventions from the RaDIANT team.
